# CircRNA expression profile and functional analysis in testicular tissue of patients with non-obstructive azoospermia

**DOI:** 10.1186/s12958-019-0541-4

**Published:** 2019-11-27

**Authors:** Pan Ge, Jian Zhang, Liang Zhou, Mo-qi Lv, Yi-xin Li, Jin Wang, Dang-xia Zhou

**Affiliations:** 1 Department of Pathology, Medical School, Xi’an Jiaotong University, Xi’an, 710061 China; 2 Research Center of Reproductive Medicine, Medical School, Xi’an Jiaotong University, Xi’an, 710061 China; 3 Reproductive Center Medicine, Maternal and child care Hospital of Shaanxi Province, Xi’an, 710063 China; 4 Obstetrics and Gynecology Department, Xi’an Angel Women’s & children’s Hospital, Xi’an, 710077 China; 5 Obstetrics and Gynecology Department, Maternal and child care Hospital of Shaanxi Province, Xi’an, 710063 China

**Keywords:** CircRNA, Spermatogenesis, Non-obstructive azoospermia (NOA), Microarray, Bioinformatics analyses

## Abstract

**Background:**

Non-obstructive azoospermia (NOA) is a multifactorial disorder whose molecular basis remains largely unknown. Circular RNAs (CircRNAs), a novel class of endogenous RNAs, have been recognized to play important roles in many biological processes. However, little is known about the expression patterns and functions of circRNAs in human testes involved in NOA.

**Methods:**

In this study, the testicular circRNA expression profile were explored in NOA patients and the controls by high-throughput circRNA microarray. Real-time quantitative reverse transcription polymerase chain reaction (qRT-PCR) was performed to confirm the microarray data. Bioinformatics analyses including the circRNA/miRNA/mRNA interaction network, Gene Ontology (GO) and Kyoto Encyclopedia of Genes and Genomes (KEGG) pathway analysis were used to predict the functions of differentially expressed circRNAs.

**Results:**

A total of 368 differentially down-regulated and 526 up-regulated circRNAs were detected in NOA patients. These findings have been verified by qRT-PCR on 6 selected circRNAs. Among these differentially expressed circRNAs, the hsa_circRNA_0023313 was obviously up-regulated in testicular tissue of NOA patients. The most likely potential target miRNA for hsa_circRNA_0023313 include hsa-miR-520d-3p, hsa-miR-373-3p, hsa-miR-372-3p, hsa-miR-302c-3p and hsa-miR-130b-5p. Function analysis indicated that hsa_circRNA_0023313 was ubiquitin-protein transferase activity and chromatin binding. KEGG analysis revealed that the top five pathways related to hsa_circRNA_0023313 were endocytosis, meiosis, FoxO signaling pathway, ubiquitin mediated proteolysis and AMPK signaling pathway.

**Conclusions:**

This is the first report that the testicular circRNA expression profile is altered in NOA patients indicating circRNAs might play important roles in regulating spermatogenesis and be potential biomarkers for the diagnosis and treatment of NOA.

## Background

Infertility is a worldwide reproductive health problem that affects an estimated 70 million people globally [1]. The world Health Organization estimate that 10–15% of couples struggle with infertility issues and male factors account for about half of all infertility cases [2, 3] Unfortunately, nearly 60–75% of male infertility is unexplained or idiopathic, since the molecular mechanisms underlying the defects remain unknown [4, 5]. Non-obstructive azoospermia (NOA) is the most severe manifestation of male infertility which spermatogenesis process is disrupted [6, 7], it affects 1% of males and 10% of those who seek fertility assistance [8]. It also demonstrated that NOA accounts for approximately 60% azoospermia in which spermatogenesis process is inactive and thus sperm cells are not generated [9]. Up to now, NOA is a multifactorial disorder whose molecular basis remains largely unknown [6, 10]. Although microdissection testicular sperm extraction (micro-TESE) is the standard therapy for NOA, sperm retrieval is unsuccessful in approximately 50% of patients [11]. Therefore, the challenge is to elucidate the precise molecular mechanisms involved in spermatogenesis process and to discover the effective diagnostic markers or therapeutic targets for NOA patients.

Circular RNAs (CircRNAs) are a novel type of endogenous RNAs featuring stable structure and high tissue-specific expression [12]. Unlike linear RNAs, circRNAs form a covalently closed continuous loop, which allows circRNAs to resist the degradation and are highly represented in the eukaryotic transcriptome [13]. CircRNAs are much more stable and conserved than linear RNAs and therefore might be involved in more abundant functions. Research has revealed that circRNAs can function as miRNA sponges, regulators of splicing and transcription, and modifiers of parental gene expression [14]. CircRNAs have been considered important biological regulators for understanding the molecular mechanisms of disease and identifying effective diagnostic biomarkers or therapeutic targets [15]. Recently, circRNAs are reported to be involved in the development of many diseases such as cardiovascular diseases and various cancers [15–19]. However, so far, to our knowledge, little is known about the expression and function of circRNAs in male infertility.

Therefore, the current study aimed to investigate the expression profile and functions of circRNAs in NOA patients. Bioinformatics analysis were also used to identify the circRNA/miRNA/mRNA interaction network, biological process and signal pathways. These results may provide potential targets for the development of novel diagnostic and therapeutic strategies against NOA.

## Materials and methods

### Patients and samples

The protocol was fully approved by the Institutional Medical Ethics Committee of Xi’an Jiaotong University. The purpose of this study was explained to all subjects, and written informed consent forms were obtained from all subjects. NOA patients were selected from couples attending the infertility clinic in reproductive center of Northwest women and children Hospital who had a history of infertility of ≥12 months. Three times semen analyses were carried out after 3–7 days of sexual abstinence. Patients with chronic diseases, hypoandrogenism, hypogonadism, history of pelvic/spinal injuries, karyotype abnormalities and microdelections of AZF region on Y chromosome were excluded. According to the World Health Organization (WHO) 2010 guidelines, all NOA patients were diagnosed by detecting three times semen samples without spermatozoa in the ejaculate including high-speed centrifugation of the entire pellet [20–23].

Finally, testicular samples were obtained from 50 patients with NOA (ages 25–46 years). An ideal normal control should consist of volunteers of known fertility, but difficulties in acquiring testicular samples makes it impractical. Therefore, 50 patients (ages 25–40 years) with obstructive azoospermia (OA) whose testicular histopathological examination demonstrated normal spermatogenesis were used as controls. Of which, 3 NOA patients whose testicular histopathological examination showed early maturation arrest and 3 controls were further used for circRNA microarray labeling and hybridization.

### RNA extraction and quality control

Total RNA was extracted from testicular biopsy tissues with TRIzol reagent according to the manufacturer’s instructions (Invitrogen, Carlsbad, California, USA). In order to reduce the inter-group difference, we mixed the three testicular tissue samples in NOA and the control group respectively for subsequent circRNA microarray labeling and hybridization. The RNA quantification and quality was examined by using the Nanodrop ND-1000 spectrophotometer. RNA integrity and gDNA contamination was tested by denaturing agarose gel electrophoresis.

### CircRNA microarray labeling and hybridization

The sample preparation and microarray hybridization were performed based on the Arraystar’s standard protocols provide by KANGCHENG Inc. (Shanghai, China). Firstly, total RNAs of 2 groups were digested with Rnase R (Epicentre, Inc.) to remove linear RNAs and enrich circular RNAs respectively. Secondly, the enriched circular RNAs were amplified and transcribed into fluorescent cRNA utilizing a random priming method (Arraystar Super RNA Labeling Kit; Arraystar). Thirdly, the labeled cRNAs were hybridized onto the Arraystar Human circRNA Array (8x15K, Arraystar). Finally, after having washed the slides, the arrays were scanned by the Agilent Scanner G2505C.

### Microarray data collection and analysis

Briefly, acquired array images were analyzed by using Agilent Feature Extraction software (version 11.0.1.1). Quantile normalization and subsequent data processing were performed using the R software package. Differentially expressed circRNAs with statistical significance between two groups were explored by Scatter Plot filtering. Differentially expressed circRNAs between samples were identified through Fold Change filtering. Hierarchical Clustering was performed to show the distinguishable circRNAs expression pattern among samples.

### Validation of circRNA by qRT-PCR

Real-time quantitative reverse transcription polymerase chain reaction (qRT-PCR) was performed to confirm the circRNA microarray data. 6 differentially expressed circRNAs (including 3 up-regulated and 3 down-regulated) were selected for qRT-PCR experiments in 50 pairs of fresh frozen testicular tissue samples (50 from NOA and 50 from OA). Specific primers designed for circRNAs were listed in Table 1. The primers were synthesized by Tsingke Biotech Ltd. (Beijing, China).

**Table 1:** Primer sequences

CircBase ID.	F/R	Primer Sequence	size (bp)
hsa_circ_0058058	F	5′-GCAGAGCTCCGAGAGTAAGG-3′	89
	R	5′-AGGCCGGTTTTGTCAGAGAC-3′	
hsa_circ_0008045	F	5′-GAGCCAGGACAAGACTCTCAA-3′	97
	R	5′-ATTCAGCAGTTGGATGCCGA-3′	
hsa_circ_0023313	F	5′-AAAACGCTACCTCGCTGCAC-3′	143
	R	5′-GGCCTTCTGCTTCGCTGAT-3′	
hsa_circ_0061817	F	5′-ACTGGTGAGGAACATCCACG-3’	91
	R	5′-AAGAACTGAATAGCCTGGCCC-3’	
hsa_circ_0002023	F	5′-CCAGCCCCAAAGAGTCAACTAA-3’	164
	R	5′-TCCATCGAGAAGGTCCACGAA-3’	
hsa_circ_0008533	F	5′-CACACCTGGACAGTCAGTTTCT-3’	125
	R	5′-ATCAGCTCCTCCAGCTCATCT-3’	
GADPH	F	5′-GCACCGTCAAGGCTGAGAAC-3’	138
	R	5′-TGGTGAAGACGCCAGTGGA-3’	

Firstly, total RNA from testicular samples was prepared using MiniBEST Universal RNA Extraction Kit (Takara, Japan) according to the manufacturer’s protocol. Secondly, total RNA was reverse transcribed into cDNA by using the HiFiScript cDNA Synthesis Kit (CWBIO, China) in a 20 μl reaction volume. Thirdly, real time PCR was performed on the Bio CFX Connect real-time PCR analyzer (Bio-Red, USA) by using the UltraSYBR Mixture (High ROX) (CWBIO, China). In brief, the total volume of 10 μl PCR reactions was prepared by mixing 5 μl UltraSYBR Mixture (2×), 0.3 μl each forward and reverse primer and 10 ng cDNA. The reaction conditions were as follows: initial incubation at 95 °C for 10 min, followed by 40 cycles of 10s denaturation at 95 °C, 30s annealing at 57 °C and 32 s extension at 72 °C. All of the experiments performed in triplicate, and the average Ct value was used to calculate the relative expression of circRNA through the comparative 2^-△△Ct^ method.

### CircRNA/miRNA interaction and circRNA/miRNA/mRNA regulatory networks analysis

To identify the potential functions of selected circRNAs, the circRNA/miRNA interaction was predicted using Arraystar’s home-made miRNA target prediction software based on miRanda [24] and TargetScan (http://www.targetscan.org) [25]. The differentially expressed circRNA were annotated in detail using the circRNA/ miRNA interaction information. In addition, the circRNA/miRNA/mRNA regulatory networks were further predicted according to the target genes of circRNA targeting miRNAs by starBase v2.0 (http://starbase.sysu.edu.cn/) [26] and miRDB (http://mirdb.org) [27].

### Bioinformatics analysis

Based on DAVID 6.8 (https://david.ncifcrf.gov/home.jsp), we conducted the Gene Ontology (GO) and Kyoto Encyclopedia of Genes and Genomes (KEGG) analysis. GO analysis was used to identify the functional roles of circRNA-targeting genes in terms of cellular components, biological processes and molecular functions. KEGG analysis was performed to explore the pathways related to circRNA-targeting genes.

### Statistical analysis

All data is described as mean ± standard deviation (SD). All statistical analyses were carried out using SPSS statistical software version 18.0 (SPSS, Chicago, USA), and *P* < 0.05 was considered statistically significant. CircRNA expression profiles in testicular tissue samples of the NOA and control group were analyzed by using paired t test. CircRNAs that demonstrate fold changes (≥2) were selected as being significantly differentially expression, and the false discovery rate (FDR) was calculated to correct the *P* value of microarray analysis results. Correlations between the relative expression of circRNAs and their ceRNA were evaluated by Pearson’s correlation method.

## Results

### Differential expression of circRNAs between the control and NOA testes

**Fig. 1:**
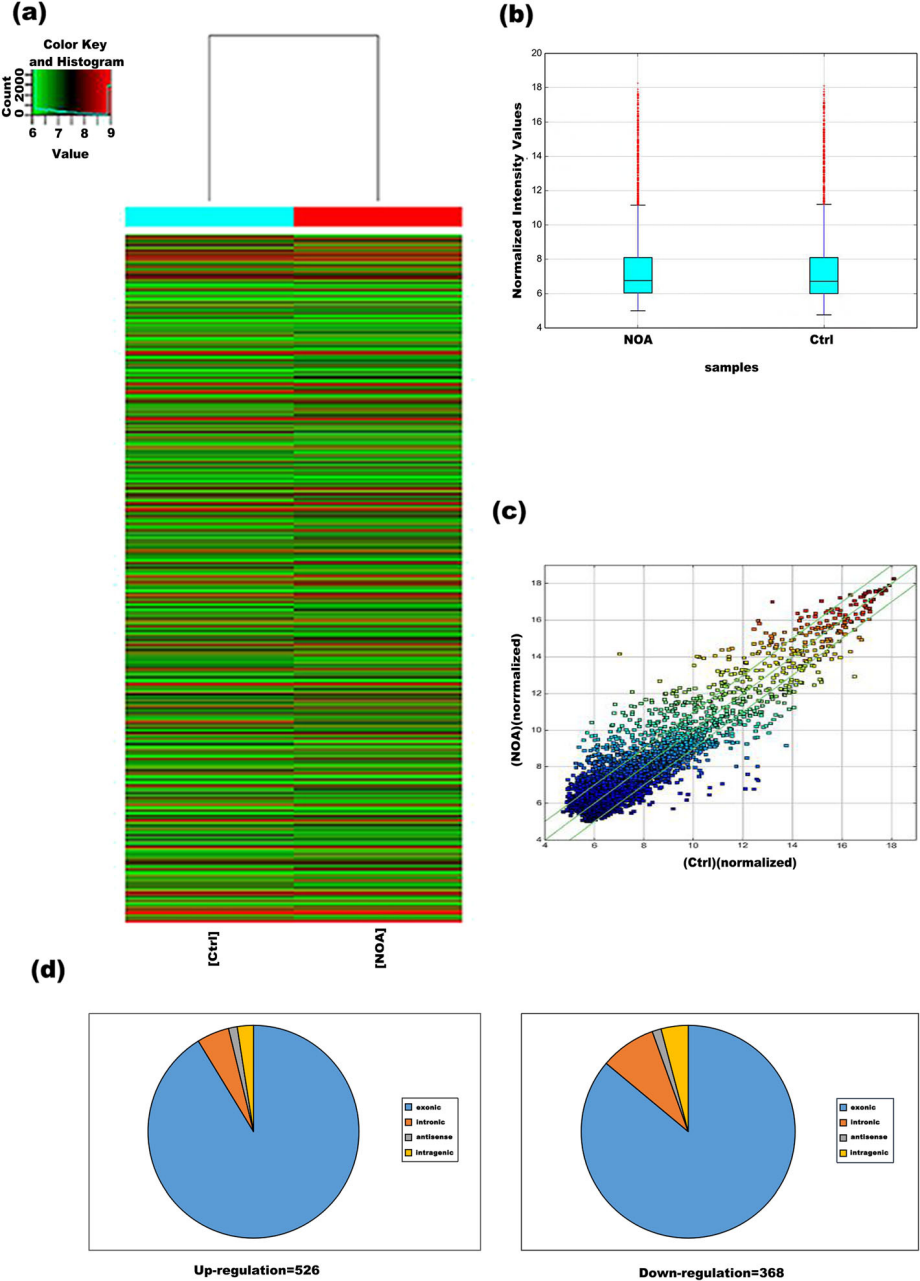
Analysis of differentially expressed circRNA in testicular tissue of NOA patients. **a** Hierarchical clustering picture of all expressed circRNAs. “red color” represents high relative expression, and “green color” represents low relative expression. **b** Box plots show that the distributions of circRNAs in the control and NOA group samples were nearly the same after normalization. **c**The scatter plots showed the variation of circRNA expression between the NOA and the control. The circRNAs in the located above the top green line and below the bottom green line indicated more than a 2.0-fold change of circRNAs. **d** The differentially expressed circRNAs based on the genomic origin were shown in the pie charts

Hierarchical clustering picture revealed the circRNA expression profile in testicular tissues of NOA patients and the control (Fig. 1a). Box plots show that the distributions of circRNAs in both NOA and the control were nearly the same after normalization (Fig. 1b). The scatter plots showed the variation of circRNA expression between the NOA and the control group (Fig. 1c). The values of X and Y axes in the scatter-plot are the normalized signal values of the samples (log2 scaled) or the averaged normalized signal values of groups of samples (log2 scaled). The green lines are Fold Change Lines. The circRNAs above the top green line and below the bottom green line indicated more than 2.0 fold change of circRNAs between the two compared samples. CircRNAs were considered to have significantly differential expression if they were up- or down-regulated at least two-fold.

A total of 4169 human circRNAs were detected. Of them, 526 human circRNAs were identified as up-regulated and 368 were down-regulated in testicular tissue of NOA patients compared to the controls (aFC > 2.0 and *P* < 0.05). According to the genomic origin of human circRNAs, the classification of the differentially expressed circRNAs was summarized in pie chart (Fig. 1d). Most of them belong to exonic circRNAs. In detail, the 526 up-regulated circRNAs consisted of 479 exonic, 26 intronic, 8 antisense and 13 intragenic. In addition, the 368 down-regulated circRNAs included 316 exonic, 31 intronic, 6 antisense and 15 intragenic (Fig. 1d).

### Validation of mircroarray data by using qRT-PCR

**Fig. 2:**
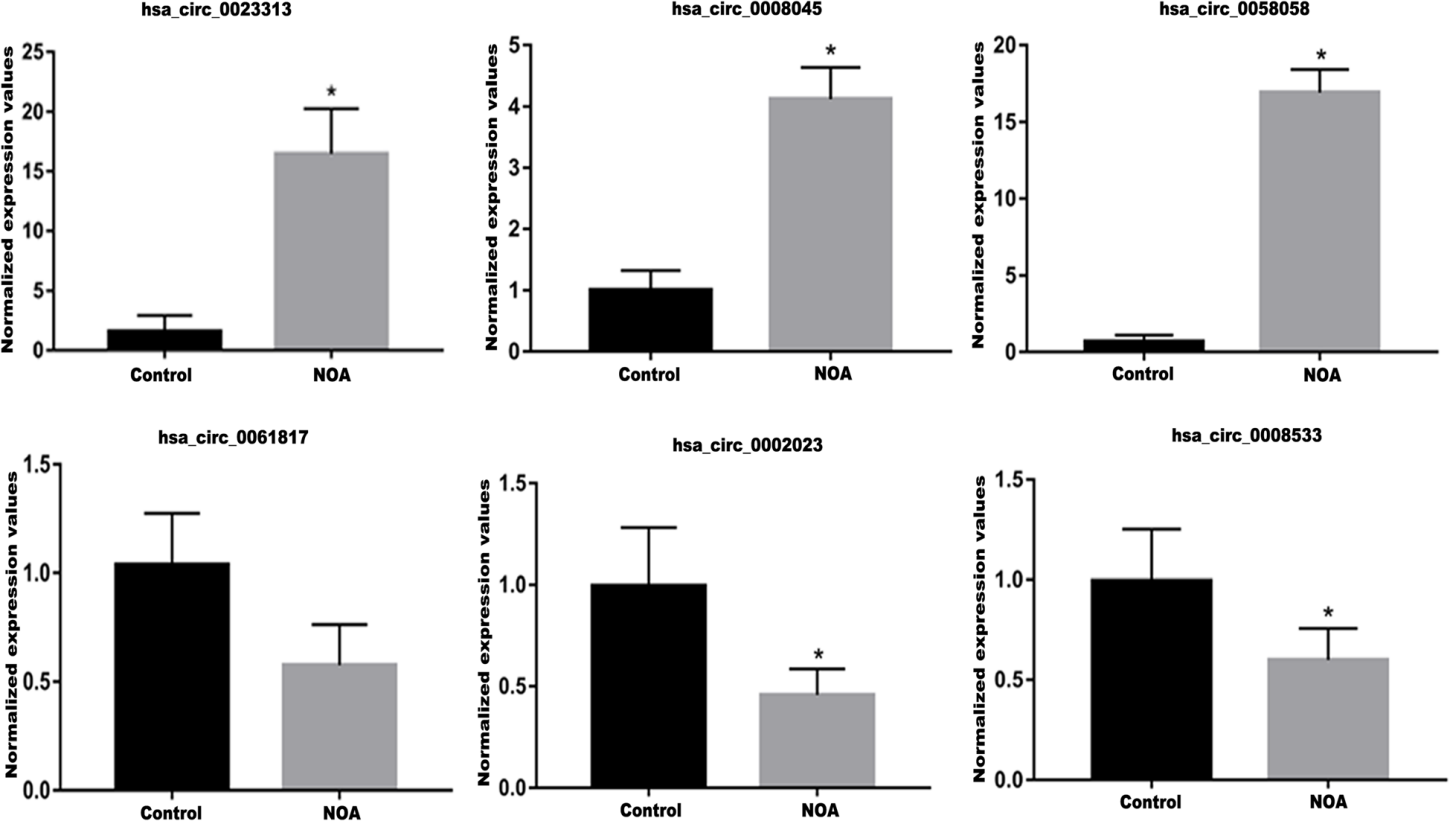
Confirmation of circRNA microarray data by qRT-PCR. The qRT-PCR analysis confirmed the circRNA microarray data. Hsa_circ_0023313 (Control 1.30 ± 1.33, NOA 16.46 ± 2.81, *P* = 0.002), hsa_circ_0008045 (Control 1.00 ± 0.32, NOA 4.12 ± 0.51, *P* = 0.00035) and hsa_circ_0058058 (Control 0.98 ± 0.43, NOA 16.93 ± 1.48, *P* = 0.0004) were up-regulated. Hsa_circ_0061817 (Control 1.04 ± 0.24, NOA 0.58 ± 0.19, *P* = 0.061), hsa_circ_0002023 (Control 1.00 ± 0.29, NOA 0.46 ± 0.13, *P* = 0.01), and hsa_circ_0008533 (Control 0.99 ± 0.26, NOA 0.60 ± 0.16, *P* = 0.012) were down-regulated in NOA patients when compared with the control. (*P < 0.05, compared with the control)

To confirm the circRNA microarray results, qRT-PCR analysis was performed on 6 randomly selected differentially expressed circRNAs, including 3 up-regulated circRNAs (hsa_circ_0058058, hsa_circ_0008045 and hsa_circ_0023313) and 3 down-regulated circRNAs (hsa_circ_0061817, hsa_circ_0002023, and hsa_circ_0008533) in control and NOA group testicular tissue samples. The results indicated that the expression patterns of selected circRNAs were in consistent with microarray data (Fig. 2), in which hsa_circ_0023313 (Control 1.30 ± 1.33, NOA 16.46 ± 2.81, *P* = 0.002), hsa_circ_0008045 (Control 1.00 ± 0.32, NOA 4.12 ± 0.51, *P* = 0.00035) and hsa_circ_0058058 (Control 0.98 ± 0.43, NOA 16.93 ± 1.48, *P* = 0.0004) was up-regulated, and hsa_circ_0061817 (Control 1.04 ± 0.24, NOA 0.58 ± 0.19, *P* = 0.061), hsa_circ_0002023 (Control 1.00 ± 0.29, NOA 0.46 ± 0.13, *P* = 0.01), and hsa_circ_0008533 (Control 0.99 ± 0.26, NOA 0.60 ± 0.16, *P* = 0.012) was down-regulated in NOA patients, compared with the control group.

### CircRNA/miRNA interaction analysis

It has been demonstrated that circRNAs function as miRNA “sponges” that competitively suppress miRNA activity and further regulate the gene expression. To find the potential circRNA/miRNA interaction in NOA, one confirmed circRNA (hsa_circRNA_0023313) was selected for further bioinformatics analysis and prediction.

**Fig. 3:**
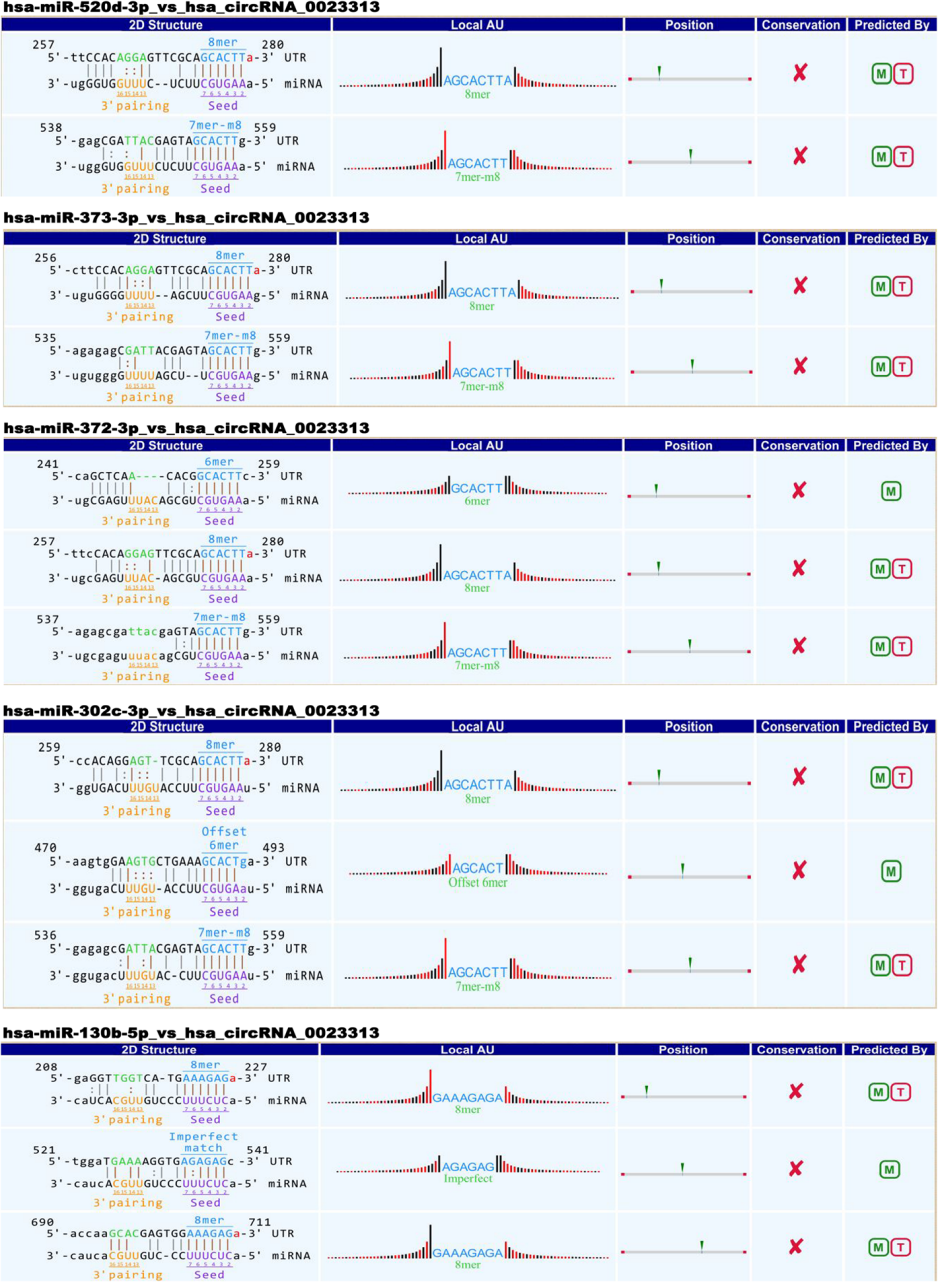
Prediction of circRNA/miRNA interaction information of hsa_circRNA_0023313. The results of hsa_circRNA_0023313 bound to sites of hsa-miR-520d-3p, hsa-miR-373-3p, hsa-miR-372-3p, hsa-miR-302c-3p and hsa-miR-130b-5p in 3′-UTR

For hsa_circRNA_0023313, the most likely potential target miRNAs are hsa-miR-520d-3p, hsa-miR-373-3p, hsa-miR-372-3p, hsa-miR-302c-3p and hsa-miR-130b-5p. The sequence analysis of miRNA response elements (MREs) are shown in Fig. 3. “The 2D structure” demonstrated the MRE sequence, the target miRNA seed type and the 3′ pairing sequence. The “Local AU” showed the AU content 30 nt upstream and downstream seed sequence. The red bars represent A/U and high accessibility, while the black bars represent G/C and low accessibility of the seed. Furthermore, the accessibility extent is demonstrated by the height of the bar. The “Position” stands for the most likely relative MRE position on the linear presentation of hsa_circRNA_002313.

### Prediction of circRNA/miRNA/mRNA interaction network

**Fig. 4:**
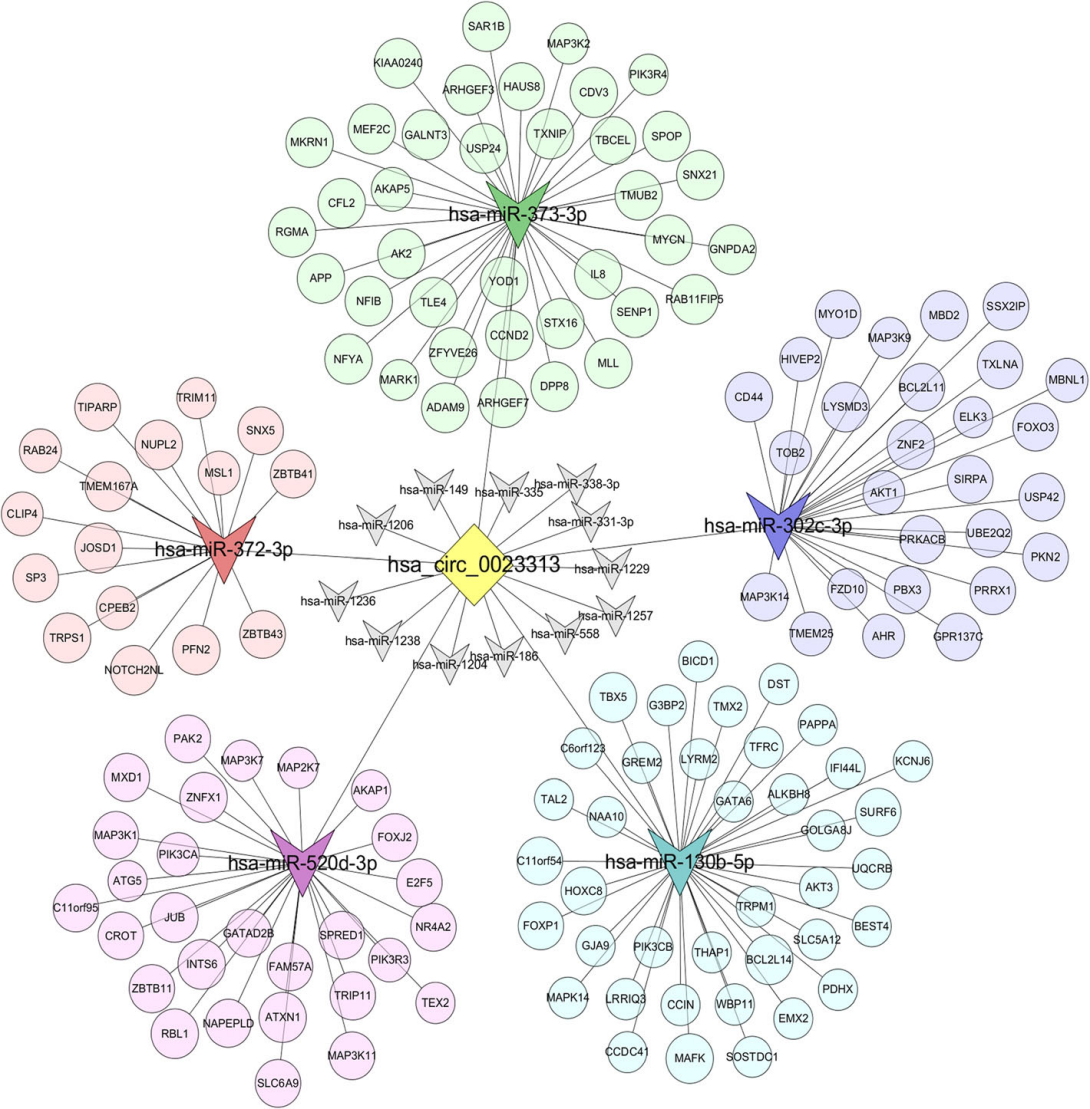
CircRNA/miRNA/mRNA interaction network diagram based on the predicted target genes of hsa_circRNA_0023313-targeting miRNAs. The yellow square in the center stands for the hsa_circRNA_0023313. The triangles in different color represent potential target miRNAs for hsa_circRNA_0023313. The different color round stands for the potential corresponding target genes (mRNA) of hsa_circRNA_0023313-targeting miRNAs

The circRNA/microRNA/mRNA interaction network diagram (Fig. 4) based on the predicted target genes of hsa_circRNA_0023313-targeting miRNAs (including hsa-miR-520d-3p, hsa-miR-373-3p, hsa-miR-372-3p, hsa-miR-302c-3p and hsa-miR-130b-5p) was drawn by Cytoscape (https://cytoscape.org/) [28].

**Fig. 5:**
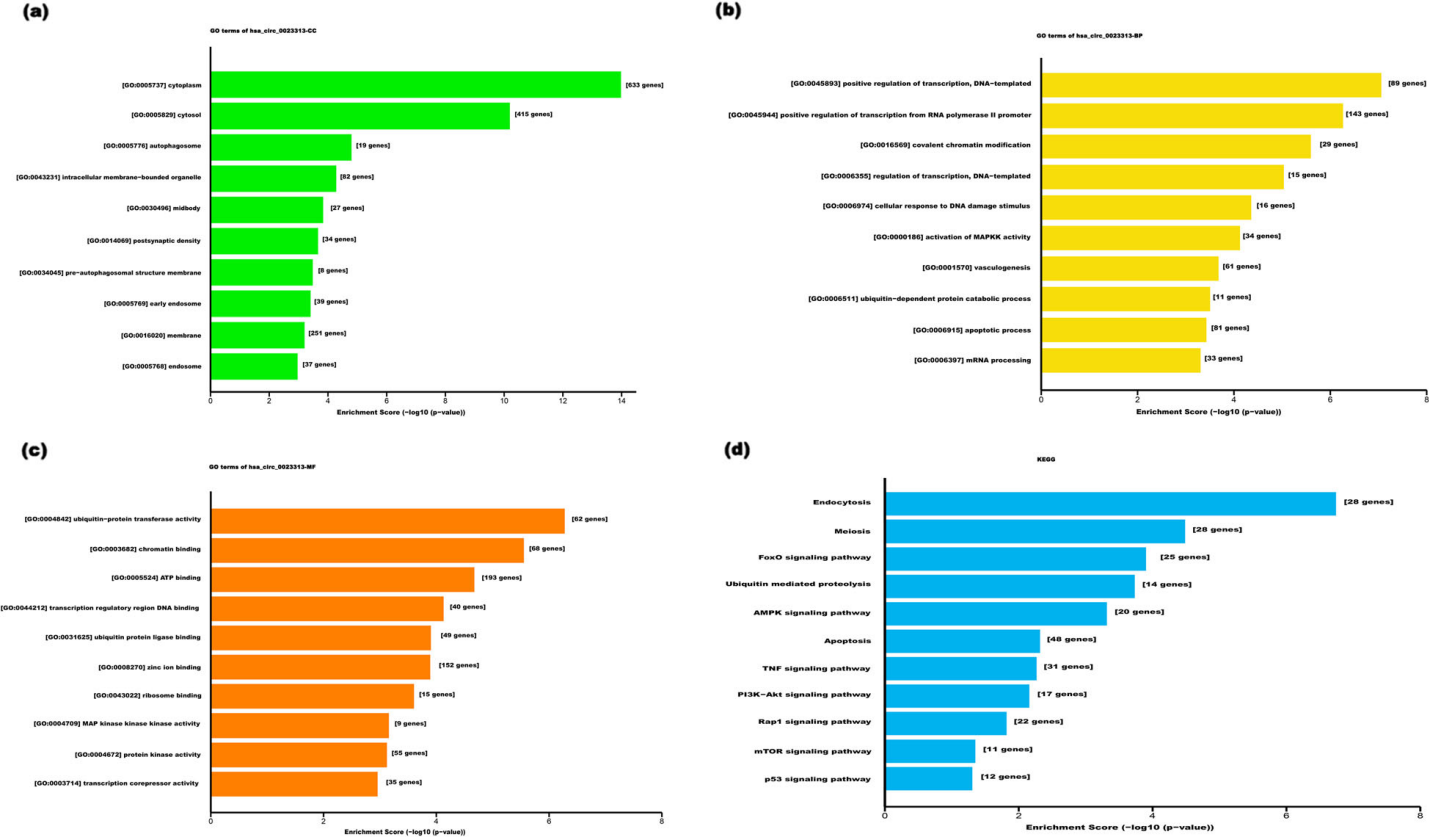
Go analysis and KEGG pathway analysis for has_circ_0023313. **a** Cellular component analysis for has_circ_0023313 targeting genes; **b** Biological process analysis for has_circ_0023313 targeting genes; **c** Molecular function analysis for has_circ_0023313 targeting genes; **d** KEGG pathway analysis for has_circ_0023313

### Go analysis and KEGG pathway analysis

Go analysis and KEGG pathway analysis were used to predict the potential biological functions of hsa_circRNA_0023313.

As shown in Fig. 5, for hsa_circRNA_0023313, the cellular component analysis revealed that its target genes were mainly involved in cytoplasm, cytosol and autophagosome and autophagosome (Fig. 5a). The biological process analysis showed its target genes were mainly involved in positive regulation of transcription, DNA-templated, positive regulation of transcription from RNA polymerase II promoter and covalent chromatin modification etc (Fig. 5b). Moreover, the molecular function analysis indicated that hsa_circRNA_0023313 was mainly involved in ubiquitin-protein transferase activity, chromatin binding and ATP-binding and so on (Fig. 5c).

KEGG analysis revealed that the top five pathways related to hsa_circRNA_0023313 were endocytosis, meiosis, FoxO signaling pathway, Ubiquitin mediated proteolysis and AMPK signaling pathway (Fig. 5d).

## Discussion

As far as we know, this is the first study to identify the comprehensive circRNAs expression pattern in testicular tissues of NOA patients. The microarray data revealed that 368 circRNAs were down-regulated and 526 circRNAs were up-regulated (aFC > 2.0 and *P* < 0.05). These findings have been confirmed by qRT-PCR assays on randomly selected circRNAs, including hsa_circ_0023313, hsa_circ_0058058, hsa_circ_0008045, hsa_circ_0061817, hsa_circ_0002023, and hsa_circ_0008533. Further systemic bioinformatics analyses including the circRNA/miRNA/mRNA interaction network, GO and KEGG pathway analysis were used to predict the functions of differentially expressed circRNAs suggesting a potential important role of circRNAs in regulating spermatogenesis.

Spermatogenesis, the transformation of spermatogonia into sperm, is a well-orchestrated and precisely-regulated biological process which is strictly regulated by phase-specific gene expression [4, 29–31]. Noncoding RNAs such as microRNAs (miRNAs), PIWI-interacting RNAs (piRNAs) and long non-coding RNAs (lncRNAs) are important post-transcriptional regulators of gene expression in multiple stages of spermatogenesis [32, 33]. CircRNAs are a novel class of conserved endogenous noncoding RNAs that could regulate gene expression [33]. It is probably the largest RNA families in human transcription [33]. Unlike linear RNAs, circRNAs form a covalently closed continuous loop and are highly represented in the eukaryotic transcriptome [13]. Thus, circRNAs are highly conserved and stability, therefore might be involved in more abundant functions [19]. Dong et al. reported that the expression of circRNAs in human testis is abundant, there are 15,996 circRNAs in normal human testis which participate the accurate gene expression regulations in spermatogenesis,, and their sequencing data were publicly in SRA database [33].

By using the circRNAs microarray and qRT-PCR analysis, we compared the circRNAs expression patterns in testicular tissues between NOA and the control. Our microarray results showed that 368 circRNAs were down-regulated and 526 circRNAs were up-regulated. To confirm the microarray data, 6 differentially expressed circRNAs were randomly selected for detection in 50 pairs of testicular tissues by qRT-PCR methods. The consistency between microarray data and qRT-PCR results further hinted that circRNAs might play important roles in regulating spermatogenesis. At the same time, we compared with the database (SRX2254041) of normal human testis circRNA deep sequence, the all of 6 circRNAs we selected were included in this database, and each of circRNA had changed [33]. Among these dysregulated circRNAs, the expression of hsa_circRNA_0023313 was dramatically enhanced in NOA patients, which indicated that it might play important roles in regulating spermatogenesis and potential biomarkers for the diagnosis, treatment of NOA.

CircRNA/miRNA/mRNA interaction network prediction provides a comprehensive understanding of the biological functions of hsa_circRNA_0023313. Our circRNA/miRNA interaction analysis demonstrated that the most likely potential target miRNA for hsa_circRNA_0023313 include hsa-miR-373-3p, hsa-miR-372-3p, hsa-miR-520d-3p, hsa-miR-302c-3p and hsa-miR-130b-5p. The study of Liu et al. showed that hsa-miR-373 and hsa-miR-372 were dysregulated in the semen of infertile males with semen abnormalities, which might be associated with semen abnormalities in infertile males [34]. In addition, Syring et al. reported that serum hsa-miR-373-3p and hsa-miR-372-3p levels were significantly increased in patients with testicular germ cell tumor compared to healthy individuals and patients with nonmalignant testicular disease [35]. The study of Hansen et al. found that the testis-specific circRNA, sex-determining region Y (Sry), serves as a miR-138 sponge, suggesting that miRNA sponge effects achieved by circRNA formation are a general phenomenon [36]. It has been showed that circRNAs function as miRNA “sponges” that competitively suppress miRNA activity and further regulate the target gene expression, and also existed in the normal human testis, thereby contributing to the development of disease [14, 33]. In the present study, hsa_circRNA_0023313 was up-regulated in NOA patients, which indicate that hsa_circRNA_0023313 might be inhibition of spermatogenesis by suppressing miRNA activity.

CircRNAs may compete with linear RNAs by binding miRNAs with miRNAs response elements (MREs), which strongly suppress miRNA activity and result in increased levels of miRNA target genes [36]. In our study, we found that putative target genes of hsa-miR-372-3p included autophagy relative gene such as RAB-24 [37] Increasing evidence suggests that autophagy play a critical role in the pathogenesis of male infertility [30, 38]. In addition, our data showed that putative target genes of hsa-miR-373-3p included ubiquitin specific protease gene such as USP24. Recent study also reported USP24 is an AR-target gene, the increased expression of the USP24 gene was associated with the initiation of sexual development, which may be involved in the regulation of spermatogenesis in mice [39]. The hsa_circRNA_0023313 may increase the expression of these target genes through competitive binding with miRNA. We speculated that hsa_circRNA_0023313 may regulate spermatogenesis by hsa_circRNA_0023313/ miR-372-3p / RAB-24 pathway and/or hsa_circRNA_0023313/ miR-373-3p / USP-24 pathway, which highly reflects the role of ceRNA regulatory network. However, the verification experiment on detailed molecular mechanisms is needed in the future.

At the same time, Go analysis and KEGG pathway analysis were used to predict the potential biological functions of hsa_circRNA_0023313. The cellular component analysis revealed that the target genes of hsa_circRNA_0023313 were mainly involved in cytoplasm, cytosol and autophagosome. The biological process analysis showed its target genes were mainly take part in positive regulation of transcription, DNA-templated and positive regulation of transcription from RNA polymerase II promoter. The molecular function analysis indicated that it mainly focuses on ubiquitin-protein transferase activity, chromatin binding and ATP-binding and so on. KEGG analysis revealed that the top five pathways related to hsa_circRNA_0023313 were endocytosis, meiosis, FoxO signaling pathway, Ubiquitin mediated proteolysis and AMPK signaling pathway. All these data strongly indicate that hsa_circRNA_0023313 might be closely related to the initiation and progression of spermatogenesis.

## Conclusions

In conclusion, this work illustrates for the first time that the comprehensive expression pattern of circRNAs in testicular tissues of NOA patients, indicating that circRNAs might play important roles in regulating spermatogenesis and it might be potential molecular targets for diagnosis and treatment of NOA. However, the exploration of molecular mechanism about the detailed role of circRNAs on spermatogenesis are still needed in the future.

## Data Availability

The dataset supporting the conclusions of this article is included within the article.
